# MGAT4EP promotes tumor progression and serves as a prognostic marker for breast cancer

**DOI:** 10.1080/15384047.2025.2475604

**Published:** 2025-03-11

**Authors:** Lin Zhong, Jianfeng Zhu, Jie Chen, Xuchu Jin, Liangquan Liu, Shufeng Ji, Jing Luo, Hong Wang

**Affiliations:** a Department of Breast Surgery, Sichuan Provincial People’s Hospital, School of Medicine, University of Electronic Science and Technology of China, Chengdu, Sichuan, China; b Department of General Surgery, Zhujiang Hospital of Southern Medical University, Guangzhou, Guangdong, China; c School of Pharmacy, Sun Yat-sen University, Guangzhou, Guangdong, China

**Keywords:** Breast cancer, MGAT4EP, tumor progression, poor prognosis

## Abstract

Breast cancer remains a global health challenge with varied prognoses despite treatment advancements. Therefore, this study explores the pseudogene MGAT4EP as a potential biomarker and therapeutic target in breast cancer. Using TCGA data and bioinformatics, MGAT4EP was identified as significantly overexpressed in breast cancer tissues and associated with poor prognosis. Multivariate Cox regression confirmed MGAT4EP as important prognostic factor. A clinical prediction model based on MGAT4EP expression showed high accuracy for 1-, 3-, and 5-year survival rates and was translated into a nomogram for clinical application. Functional studies revealed that silencing MGAT4EP *via* siRNA promoted apoptosis, inhibited migration and invasion in breast cancer cells. RNA-seq, GSEA, and GO analyses linked MGAT4EP to apoptosis and focal adhesion pathways. Notably, knock down of MGAT4EP significantly suppressed tumor growth and metastasis in xenograft and lung metastasis models. Taken together, these findings establish MGAT4EP as an attractive target for metastatic breast cancer and provide a potential a promising therapeutic target for breast cancer treatment.

## Introduction

Breast cancer is one of the most common malignancies among women worldwide and continues to pose a severe threat to women’s health and lives.^1^ According to the latest statistics, the global incidence of breast cancer has shown a marked increase from 2000 to 2020.^2^ In 2022, more than 2.3 million new breast cancer cases were diagnosed, accounting for approximately 11.6% of all new cancer cases.^3^ Despite substantial progress in early diagnosis, surgical treatments, chemotherapy, and targeted therapies, which have contributed to a decrease in breast cancer mortality,^4^ approximately 670,000 women globally succumbed to breast cancer in 2022, accounting for 6.9% of all cancer-related deaths.^3^ Improving the prognosis of breast cancer patients remains a significant challenge.^5^ Therefore, the active exploration of new diagnostic and therapeutic strategies is crucial.^6^ The identification of new prognostic biomarkers and the development of targeted therapies for breast cancer hold great clinical significance in improving patient outcomes.^7–10^

In recent years, the rapid advancement of high-throughput sequencing technologies, combined with the continuous development of bioinformatics methods, has led to unprecedented progress in our understanding of tumor genomics.^11–13^ As part of this process, pseudogenes – sequences once regarded as “genomic junk” – have gained increasing attention from researchers.^14^ As studies progress, these genetic materials, previously considered “useless,” may emerge as valuable tools for disease diagnosis, prognosis, and treatment.^15–17^

MGAT4EP, a newly identified pseudogene, is localized in the cell nucleus and interacts with FOXA1, a key regulatory factor in breast cancer. MGAT4EP enhances the promoter-binding ability of FOXA1, which, in turn, upregulates the expression of the oncogenic transcription factor FOXM1.^18^ This indicates that MGAT4EP may play a critical role in the progression of breast cancer. In this study, we first conducted a bioinformatics analysis by mining breast cancer-related transcriptomic and clinical data from the TCGA database.^19^ After rigorous data filtering and analysis, we identified the pseudogene MGAT4EP as an independent predictor of poor prognosis in breast cancer. The predictive model based on MGAT4EP expression was found to effectively assess patient prognosis. To further validate the function of MGAT4EP in breast cancer, we conducted RNA sequencing as well as *in vitro* and *in vivo* experiments to investigate its role in cell apoptosis, migration, and invasion, and to elucidate the specific mechanisms underlying breast cancer progression.

Through this study, we aim to elucidate the critical role of MGAT4EP in breast cancer progression, providing new insights and potential targets for both prognostic prediction and therapeutic strategies in breast cancer.

## Results

### MGAT4EP is an important prognostic marker for breast cancer

**Figure 1.**
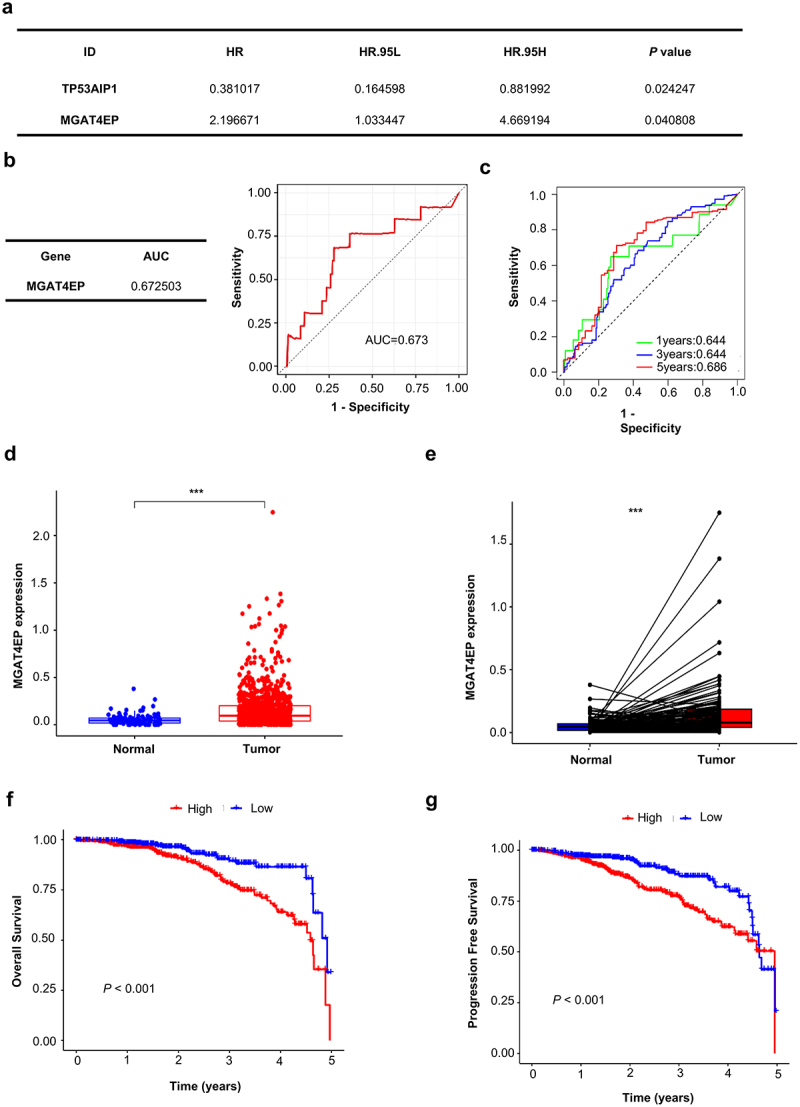
MGAT4EP is an important prognostic Marker for breast Cancer. (a) Prognostic correlation analysis identifies target genes associated with breast cancer prognosis from the TCGA database. (b) ROC curve evaluation of MGAT4EP’s predictive ability for breast cancer prognosis. (c) ROC curve analysis of MGAT4EP’s predictive ability for 1-year, 3-year, and 5-year overall survival (OS) in breast cancer patients. (d) Analysis of the expression difference of MGAT4EP between normal and tumor tissues. (e) Paired analysis confirming the expression difference of MGAT4EP between normal and tumor tissues. (f) Survival analysis comparing the 5-year OS between high and low MGAT4EP expression groups. (g) Comparison of 5-year disease-free survival (DFS) between high and low MGAT4EP expression groups. *p < 0.05, **p < 0.01, ***p < 0.001.

To identify genes associated with breast cancer prognosis, we downloaded the BRCA mRNA dataset from the TCGA database, which includes patient information from 1,171 individuals. COX regression analysis was performed to identify risk genes that influence breast cancer prognosis. The results showed that TP53AIP1 had a Hazard Ratio (HR) of 0.381 (95% CI: 0.165–0.882, *p* = .024), while MGAT4EP had an HR of 2.197 (95% CI: 1.033–4.669, *p* = .041) (Figure 1a and Supplementary Table 1).

To further validate the prognostic value of these genes in breast cancer, we conducted COX regression analysis and found that MGAT4EP exhibited predictive performance for breast cancer prognosis with an AUC > 0.6, specifically AUC = 0.673 (Figure 1b), while TP53AIP1 showed a lower predictive ability (Supplementary Figure S1a). Further analysis revealed that MGAT4EP predicted 1-year and 3-year survival rates with an AUC of 0.644, and a 5-year survival prediction with an AUC of 0.686 (Figure 1c). Differential expression analysis revealed that MGAT4EP was significantly overexpressed in breast cancer tissues compared to normal tissues (Figure 1d). Pairwise analysis of tumor and normal tissues further confirmed that MGAT4EP expression was elevated in tumor tissues, with the difference being statistically significant (Figure 1e). By stratifying breast cancer patients into high and low MGAT4EP expression groups, we found that the overall survival (OS) and progression-free survival (PFS) were significantly lower in the high-expression group compared to the low-expression group, with the difference being statistically significant (*p* < .001) (Figure 1f-g). Therefore, MGAT4EP represents a significant prognostic marker associated with poor outcomes in breast cancer.

### The expression of MGAT4EP is closely related to the clinical characteristics of breast cancer

**Figure 2.**
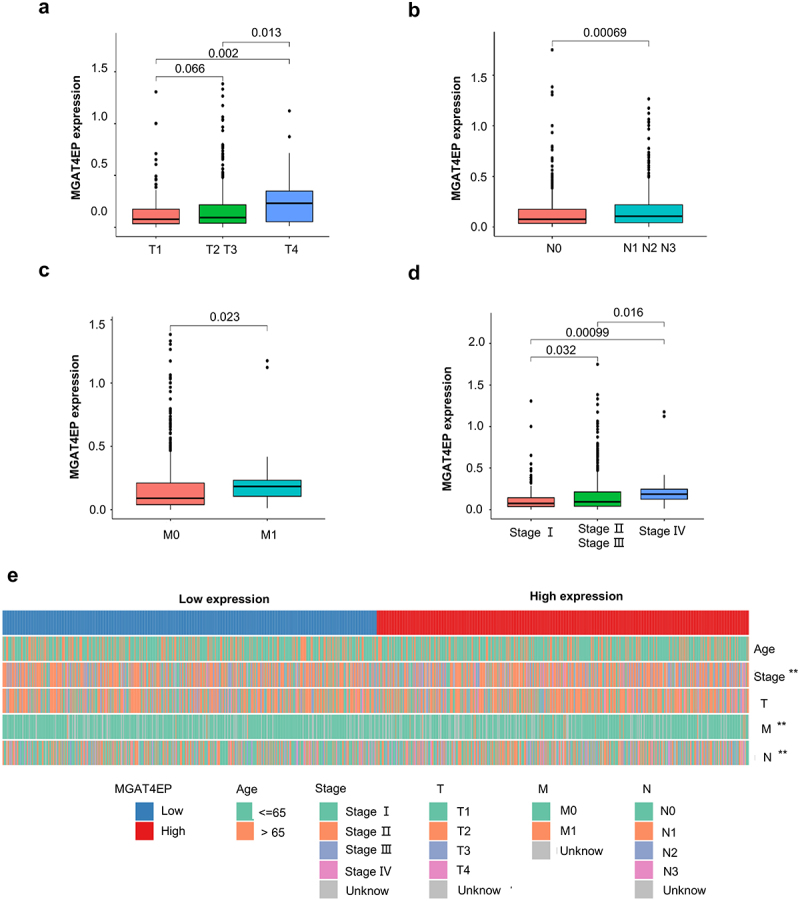
The expression of MGAT4EP is closely related to the clinical characteristics of breast Cancer.(a) Analysis of MGAT4EP expression differences in different T stages of breast cancer.(b) Comparison of MGAT4EP expression in breast cancer patients with and without lymph node metastasis.(c) Comparison of MGAT4EP expression in breast cancer patients with or without distant metastasis.(d) Analysis of MGAT4EP expression in breast cancer patients of different stages.E. Clinical feature comparison based on MGAT4EP high and low expression groups.

TNM staging is one of the most widely employed systems for predicting and evaluating the prognosis of breast cancer patients in clinical practice. In our previous study, we identified MGAT4EP as an important marker of poor prognosis in breast cancer patients. This raises the question: is MGAT4EP expression associated with the clinical characteristics of breast cancer patients? Our analysis showed that the expression of MGAT4EP was significantly higher in patients with T4 stage breast cancer compared to those with T1, T2, or T3 stages (T4 vs T1, *p* = .003; T4 vs T2/T3, *p* = .013) (Figure 2a). Lymph node metastasis is the most common type of metastasis in breast cancer. We found that MGAT4EP was highly expressed in lymph node-positive patients, with a statistically significant difference between the two groups (*p* = .00069) (Figure 2b). Distant metastasis i also plays a critical role in influencing the prognosis of breast cancer patients. Notably, we found that MGAT4EP was highly expressed in patients with metastatic breast cancer showing a significant difference compared to normal breast tissue (*p* = .023) (Figure 2c). Thus, MGAT4EP is associated with both lymph node and distant metastasis in breast cancer patients.

*p < 0.05, **p < 0.01, ***p < 0.001.

AJCC staging, which is based on TNM classification, is an important clinical staging system that provides a more accurate description of patients prognosis. In this study, we found that MGAT4EP expression significantly increased with the stage of the disease. The expression levels were as follows: Stage II, III vs. Stage I *p* = .032; Stage IV vs. Stage I, *p* = .00099; Stage IV vs. Stage II, *p* = .016 (Figure 2d). Based on the median expression level of MGAT4EP, patients were divided into high-expression and low-expression groups. The results showed that the high-expression group exhibited statistically significant differences compared to the low-expression group in terms of Stage, M, and N classifications (*p* < .001) (Figure 2e). Moreover, we have also observed that the pseudogene MGAT4EP is expressed in TNBC cell lines, and its expression is significantly higher in tumor tissues compared to normal tissues (Supplementary Figure S4a-b). Therefore, MGAT4EP is strongly correlated with the clinical staging of breast cancer and represents a promising biomarker for predicting patient prognosis.

### Independent prognostic role of MGAT4EP in breast cancer: univariate and multivariate analysis

In previous studies, we confirmed that the expression of MGAT4EP is closely associated with the prognosis of breast cancer patients and correlates with the clinical staging of the disease, particularly exhibiting significantly elevated expression levels in patients with metastasis (including both lymph node metastasis and distant metastasis). However, whether MGAT4EP serves as an independent prognostic factor for breast cancer still requires further validation. When MGAT4EP expression was analyzed in conjunction with TNM and age, univariate Cox regression analysis revealed that MGAT4EP expression is a negative prognostic factor for breast cancer, with a Hazard Ratio (HR) of 4.513 (95% CI: 2.024–10.060), *p* < .001 (Supplementary Figure S2a). Further multivariate Cox regression analysis confirmed that MGAT4EP expression remained a significant factor for poor prognosis in breast cancer patients, with an HR of 2.661 (95% CI: 1.076–6.579), *p* = .034 (Supplementary Figure S2b).

**Figure 3.**
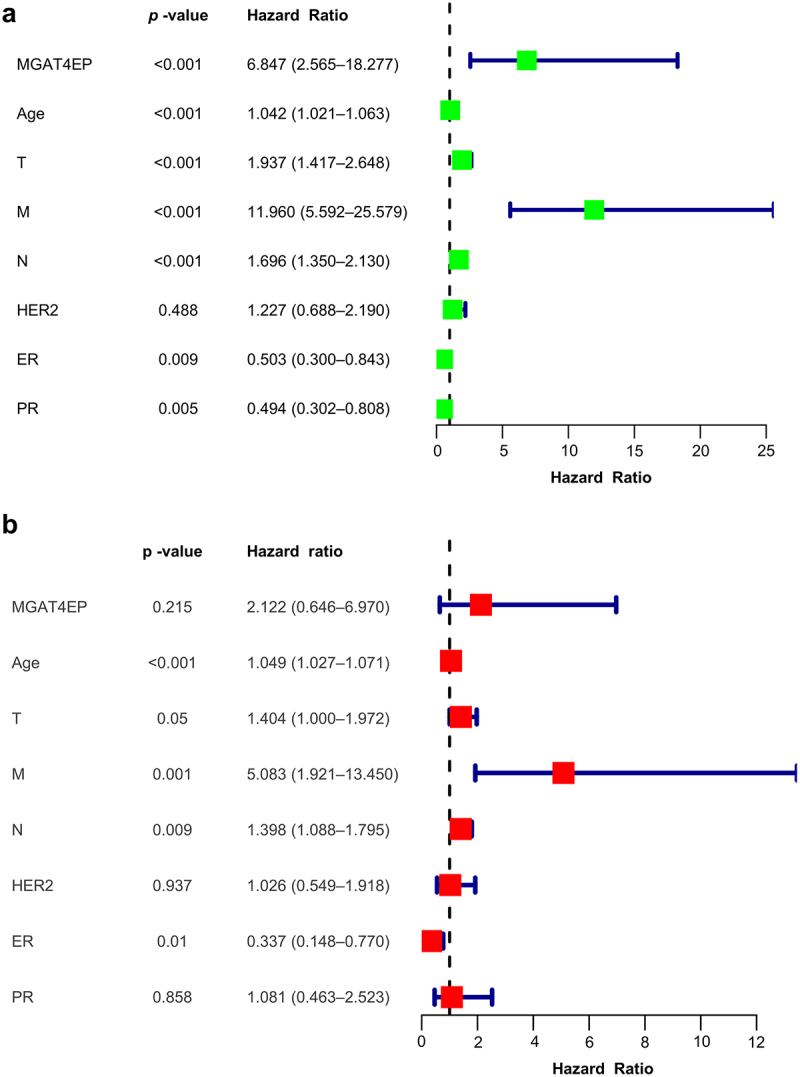
Independent prognostic role of MGAT4EP in breast cancer: univariate and multivariate analysis.(a) Univariate analysis of MGAT4EP combined with age, tumor size, lymph node metastasis, distant metastasis, and the status of ER, PR, and HER2, presented as a forest plot.(b) Multivariate analysis of MGAT4EP combined with age, tumor size, lymph node metastasis, distant metastasis, and the status of ER, PR, and HER2, also presented as a forest plot. *p < .05, **p < .01, ***p < .001.

After further analysis of the expression of MGAT4EP in breast cancer in conjunction with the status of ER, PR, and HER2, univariate analysis revealed a p-value of < 0.001 and an HR of 6.847 (95%CI:2.565–18.277) (Figure 3a), In the multivariate analysis, age, lymph node metastasis (N) and distant metastasis (M), and ER expression status remained statistically significant (*p*  < 0.05), but the HR for MGAT4EP was, *p*  = 0.215 (Figure 3b). A possible explanation for this result is that the ER, PR, HER2 status hold a higher diagnostic value for breast cancer prognosis. Nevertheless, MGAT4EP may still act as an independent prognostic factor for breast cancer.

### Construction of a prognosis prediction model based on MGAT4EP

Based on the results of previous studies, MGAT4EP has been identified as an important prognostic factor for breast cancer. We integrated MGAT4EP expression with statistically significant parameters from multivariate analysis to construct a prognostic prediction model:

$$\mathrm{Risk}\;\mathrm{Score}=\;-0.618{\;}^{*}\mathrm{MGAT}4\mathrm{EP}\left(\mathrm{low}\right)\;+\;0.043{\;}^{*}\mathrm{Age}+\;1.603{\;}^{*}M1\;+\;0.375{\;}^{*}N1\;+\;0.902{\;}^{*}N2\;+\;1.315{\;}^{*}N3\;+\;-0.903{\;}^{*}\mathrm{ER}\left(\mathrm{Positive}\right)$$

**Figure 4.**
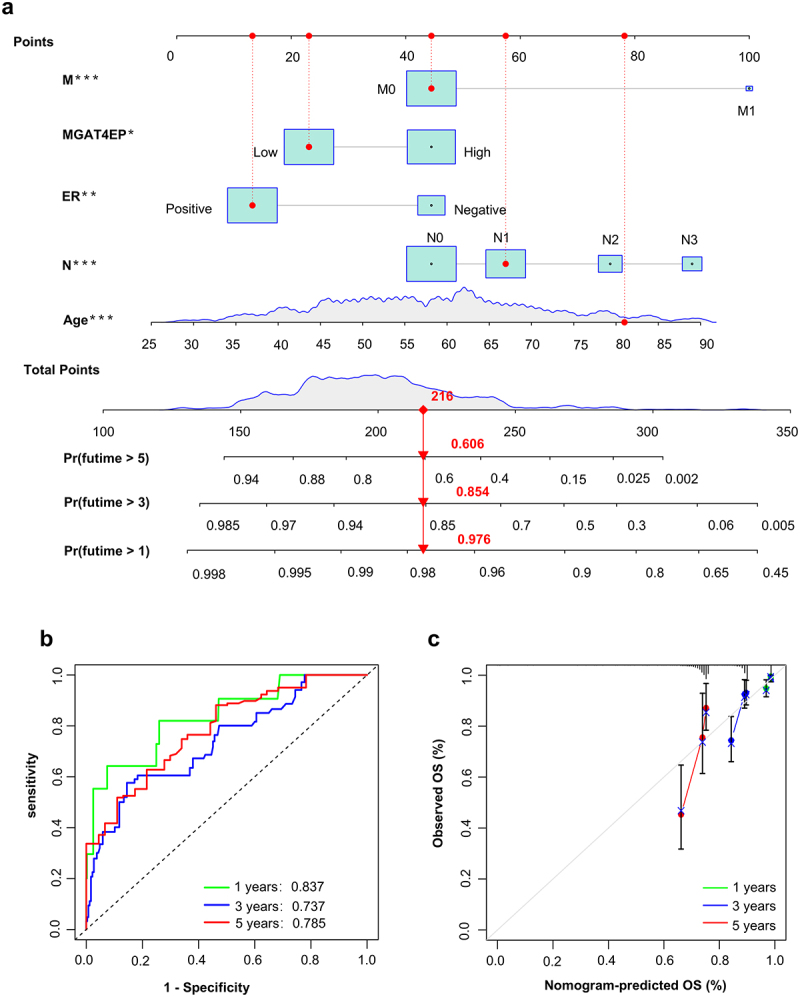
Development of a nomogram based on MGAT4EP.(a) Nomogram visually illustrating the risk prediction model structure. (b) ROC curve analysis evaluating the model’s predictive ability for 1-year, 3-year, and 5-year survival. (c) Calibration curve assessing the consistency between predicted survival probabilities and actual survival outcomes. *p < .05, **p < .01, ***p < .001.

This model incorporates five key factors: MGAT4EP expression, N and M stages, ER expression status and age. To facilitate clinical application, we developed a nomogram based on this model. The nomogram can be used to predict the 1-year, 3-year, and 5-year survival probabilities of patients by calculating the scores for each variable (Figure 4a). For example, for a patient approximately 80 years old, with N1, M0, ER positive, and low MGAT4EP expression, the corresponding scores for each factor generate a total score of 216, corresponding to survival probabilities of 0.976 for 1 year, 0.854 for 3 years, and 0.606 for 5 years, respectively. As shown in Figure 4b, the model’s prediction accuracy was 0.837 for 1-year survival, 0.737 for 3-year survival, and 0.785 for 5-year survival. The calibration curve presented in Figure 4c demonstrates that the model exhibits good predictive capability, especially for the 1-year and 3-year survival predictions. These findings highlight a significant correlation between MGAT4EP and clinical staging in breast cancer, underscoring its potential as a predictive biomarker for patient outcomes.

### MGAT4EP affects the progression of breast cancer by regulating apoptotic pathways

**Figure 5.**
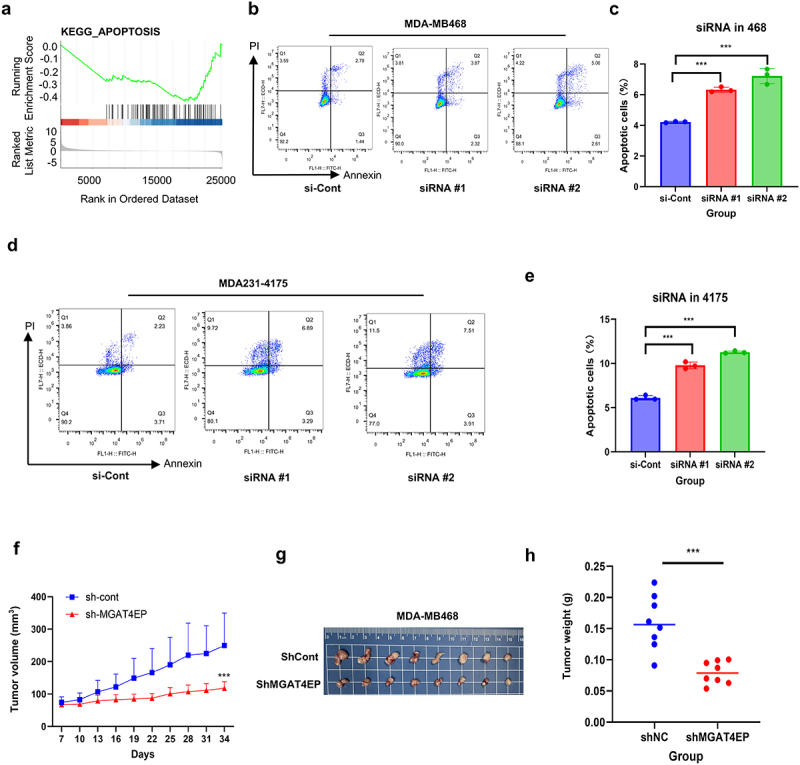
MGAT4EP Affects the Progression of Breast Cancer by Regulating Apoptotic Pathways. (a) GSEA analysis revealing the correlation between MGAT4EP affects the progression of breast cancer by regulating apoptotic Pathways.(b). GSEA analysis revealing the correlation between MGAT4EP and the apoptosis signaling pathway in breast cancer.(b-e) Flow cytometry analysis showing the increase in apoptosis in MDA-MB-468 and MDA-MB-231-4175 cells following MGAT4EP silencing by siRNA. *n* = 3 biological replicates.(f) Tumor volume changes over time in sh-nc and sh-MGAT4EP groups (*n* = 8). Mean tumor volume ± SEM.(g) Schematic diagram of tumor volumes in sh-nc and sh-MGAT4EP groups (*n* = 8).(h) Statistical analysis of tumor weight in the sh-nc and sh-MGAT4EP groups (*n* = 8). Mean tumor weight ± SEM.

The critical role of MGAT4EP in breast cancer prognosis has been confirmed. To further elucidate the mechanisms by which MGAT4EP regulates the onset and progression of breast cancer, we first performed Gene Set Enrichment Analysis (GSEA) analysis on TCGA data. The results revealed that MGAT4EP influences the development of breast cancer by modulating the APOPTOSIS signaling pathway (Figure 5a, supplementary Figure S5). Flow cytometry was used to assess the impact of MGAT4EP on apoptosis in breast cancer cells. The results showed that after siRNA transfection in MDA-MB468 and MDA231-LM2-4175 cells led to a significant increase in apoptosis (*p*  < 0.001) (Figure 5b-e). To further investigate the effect of MGAT4EP on tumor growth, we constructed a sh-MGAT4EP viral vector. After transfecting MDA-MB-468 breast cancer cells with the viral vector and establishing a cell-derived xenograft (CDX) model, we observed that knockdown of MGAT4EP significantly inhibited tumor growth (Figure 5f). Statistical analysis revealed a significant difference in tumor volume between the two groups, with this difference becoming more pronounced over time. Tumors in the sh-MGAT4EP group were significantly smaller than those in the control group (Figure 5g,h). In summary, our study demonstrates that MGAT4EP plays a pivotal role in breast cancer prognosis by regulating apoptosis and significantly inhibiting tumor growth, thereby highlighting its potential as a therapeutic target.

All data from *in vitro*experiments shown above are the mean ± SD. *p < 0.05, **p < 0.01, ***p < 0.001.

### MGAT4EP promotes breast cancer metastasis through the focal adhesion pathway

**Figure 6.**
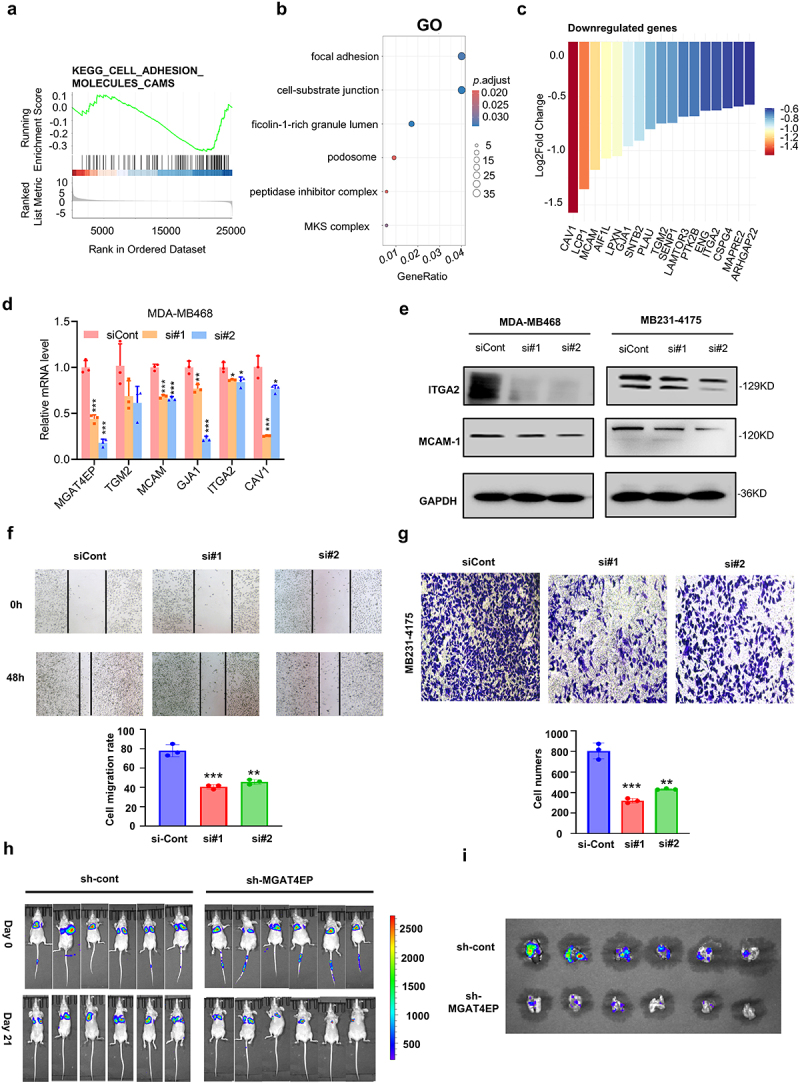
MGAT4EP promotes breast cancer metastasis through the focal adhesion Pathway.(a) GSEA analysis showing the significant correlation between MGAT4EP and the focal adhesion signaling pathway in breast cancer.(b) RNA-seq analysis and subsequent GO enrichment analysis exploring the impact of MGAT4EP silencing in MDA-MB-468 cells. (c) Downregulation of focal adhesion pathway genes following MGAT4EP silencing in MDA-MB-468 cells.(d) RT-PCR analysis of key gene expression changes in the focal adhesion pathway following MGAT4EP silencing. *n* = 3 biological replicates.(e) Western blotting (WB) detection of protein level changes of key genes in the focal adhesion pathway, further confirming the regulatory effect of MGAT4EP. Representative blots are shown. *n* = 3 biological replicates.(f) Scratch assay showing the effect of MGAT4EP silencing on the migration ability of MDA-MB-231-4175 cells. *n* = 3 biological replicates.(g) Transwell assay exploring the effect of MGAT4EP silencing on the invasion ability of MDA-MB-231-4175 cells. *n* = 3 biological replicates.(h) Lung metastasis model construction via tail vein injection of sh-nc and sh-MGAT4EP infected MDA-MB-231-4175 cells, monitored by in vivo imaging of lung metastasis tumors (*n*  = 6).(i) Postmortem analysis of experimental animals, showing the formation and distribution of lung tumors, visualized using *in vivo* imaging (*n*  = 6).

Metastasis is a critical risk factor in breast cancer. To further investigate the role of MGAT4EP in breast cancer metastasis, we conducted GSEA analysis on TCGA data, which suggested that MGAT4EP may promote breast cancer metastasis by regulating cell adhesion-related pathways (Figure 6a). Subsequently, we transfected MB231–4175 cells with siMGAT4EP or siCont and performed RNA-seq sequencing. The results showed that MGAT4EP knockdown significantly downregulated the focal adhesion pathway in MB231–4175 cells, thereby suppressed cancer cells distant metastasis (Figure 6b). Pathway-focused analysis revealed that MGAT4EP siRNA significantly downregulated the expression of key genes involved in cell-substrate junction, cell focal adhesion including CAV1, ITGA2, and MCAM (Figure 6c). Furthermore, qRT-PCR and Western blot analyses demonstrated that knockdown of MGAT4EP significantly downregulated the expression of key genes involved in the focal adhesion pathway at both the mRNA and protein levels (Figure 6d,e).

All data from *in vitro* experiments shown above are the mean ± SD. *p < 0.05, **p < 0.01, ***p < 0.001.

To further investigate whether MGAT4EP inhibits cell migration *in vitro*. We performed scratch assays and Transwell experiments, both of which demonstrated that the suppression of MGAT4EP significantly reduced the migratory and invasive capabilities of the cells (Figure 6f,g, supplementary Figure S6a). Lung metastasis is one of the most common forms of distant metastasis in breast cancer. To further investigate the effect of MGAT4EP on breast cancer metastasis, we constructed a sh-MGAT4EP viral vector and used it to infect the MB231–4175 cell line. Following tail vein injection, we established a lung metastasis model. *In vivo* imaging of the mice showed significant differences in lung metastasis between the two groups. On day 21, the extent of lung metastasis in the sh-MGAT4EP group was notably lower than that in the sh-NC group (Figure 6h). Furthermore, upon dissection on day 21, lung imaging showed that the sh-NC group exhibited significantly more metastasis compared to the sh-MGAT4EP group (Figure 6i, supplementary Figure S6b). Therefore, suppression of MGAT4EP expression significantly inhibits lung metastasis in breast cancer.

## Discussion

This study deeply provides an in-depth exploration of breast cancer-associated genes and highlights the critical role of the pseudogene MGAT4EP in breast cancer prognosis. By integrating transcriptomic and clinical data from the TCGA database, we conducted comprehensive survival analysis and differential expression analysis, employing various bioinformatics tools. Our findings, demonstrate that MGAT4EP is not only a significant prognostic factor for breast cancer but also plays a pivotal role in the description of clinical feature description, precise prognostic prediction, and the mechanisms underlying tumor metastasis mechanisms.

The application of bioinformatics to analyze large-scale public data for prognostic biomarkers in breast cancer has emerged as a new research hotspot.^20–24^ Through rigorous prognostic correlation analysis and assessments of predictive capabilities, we successfully identified MGAT4EP from the TCGA database. Our results showed that the MGAT4EP expression levels were significantly higher in breast cancer tissues compared with normal tissues, and that high expression was closely associated with poor prognosis among patients. Specifically, patients with high MGAT4EP expression had significantly shorter OS and PFS compared to the low-expression group, with highly statistically significant differences (*p* < .001). These findings suggest that MGAT4EP may serve as an important biomarker for poor prognosis in breast cancer. While previous studies have primarily focused on the relationships between coding genes and breast cancer prognosis,^25–27^ the research value of pseudogenes has gained increasing recognition in recent years.^28^ For example, studies have confirmed that pseudogenes such as ABCC6P1, TPTEP1and RPSAP52, play significant roles in breast cancer.^29–31^ Further validation through ROC curve analysis demonstrated the robust prognostic predictive capability of MGAT4EP, with an AUC of 0.672, highlighting its strong performance in predicting 1-year, 3-year, and 5-year OS.

In-depth analysis revealed a strong association between MGAT4EP expression levels and clinical staging in breast cancer, particularly among patients with lymph node metastasis and distant metastasis, where MGAT4EP expression was significantly up-regulated. Furthermore, MGAT4EP expression demonstrated a significant correlation with T stage, lymph node metastasis, distant metastasis, and AJCC staging of breast cancer (*p* < .05). These important findings reinforce the potential of MGAT4EP as a potential prognostic marker for breast cancer, assisting clinicians in identifying high-risk patients for metastasis and providing valuable insights for individualized treatment strategies.

When we combined MGAT4EP expression with TNM staging and age in the analysis, univariate and multivariate Cox regression analyses confirmed that MGAT4EP expression is an independent risk factor for breast cancer prognosis (Supplementary Figure S2). In a prognostic model that incorporated TNM stage, hormone receptor status, age, and MGAT4EP, we demonstrated effective prognostic predictive ability. Our model, which integrates common clinical indicators like estrogen receptor status, age, lymph node status, distant metastasis, and MGAT4EP expression, shows superior clinical application potential and feasibility when compared to existing models that require extensive sequencing data.^32,33^ With prediction accuracies of 0.837, 0.737, and 0.785 for 1-year, 3-year, and 5-year survival rates, respectively, our model outperforms the widely adopted TNM staging system (which has prediction accuracies of 0.66, 0.651, and 0.617 for the same time points) across all time intervals (Supplementary Figure S3). However, further external validation is needed to fully establish its reliability and accuracy.

For clinical application, we carefully designed a nomogram,^34^ that enables rapid calculation of risk scores based on the relevant indicators. The nomogram clearly shows that factors such as Age, M1 (distant metastasis status), LN+ (lymph node metastasis positive), ER negative, and high MGAT4EP expression are critical contributors to poor prognosis in breast cancer patients, which is highly consistent with the clinical TNM staging results.^35^ However, we also observed some discrepancies in assessing age-related risk within the model compared to actual clinical scenarios.^36,37^ This may be due to the assumption of a linear relationship between age and risk, while the relationship between age and breast cancer prognosis is likely more complex, particularly for younger patients (≤40) who often face a higher risk of aggressive breast cancer subtypes.^38–40^ Future studies could optimize the model by incorporating non-linear modeling or considering interactions between age and other variables to better reflect the actual impact of age on breast cancer prognosis.

Regarding the specific mechanisms of MGAT4EP in breast cancer, it is still not fully understood. Previous studies have demonstrated that MGAT4EP enhances FOXA1 binding to its promoter in luminal A tumors, leading to the upregulation of FOXM1 and promoting breast cancer growth and metastasis.^18^ To explore the biological functions of MGAT4EPmore thoroughly, we performed GSEA analysis and identified that MGAT4EP might regulate multiple key signaling pathways involved in breast cancer development, particularly those related to apoptosis and cell adhesion. Flow cytometry analysis further confirmed that suppressing MGAT4EP expression significantly increased apoptosis in breast cancer cells, suggesting that MGAT4EP may contribute to the survival of breast cancer cell survival by inhibiting apoptosis.^41^ Additionally, combining TCGA data with RNA-seq gene enrichment analysis, we found that MGAT4EP affects breast cancer cell migration and invasion by regulating the focal adhesion pathway.^42^ RT-PCR and Western blot analyses provided strong evidence, demonstrated that suppression of MGAT4EP significantly down-regulated adhesion molecules such as CAV1,^43^ ITGA2,^44^ and MCAM.^45^ This underscores the critical role of MGAT4EP’s critical role in cell migration and metastasis.

*In vivo* experiments also provided important evidence of MGAT4EP’s role in tumor growth and metastasis. In the mouse CDX model, the inhibition of MGAT4EP expression significantly slowed tumor growth, resulting in markedly smaller than in the control group. Additionally, in the lung metastasis model, suppression of MGAT4EP significantly reduced lung metastasis in mice, further validating the crucial role of MGAT4EP in breast cancer metastasis.

Although MGAT4EP lacks a complete open reading frame, its retained sequence homology to the functional MGAT4 gene family suggests the possibility of competitive regulation. Pseudogenes can act as competing endogenous RNAs (ceRNAs), binding to microRNAs (miRNAs) or RNA-binding proteins, thus modulating the stability or translation of their homologous mRNA counterparts. For instance, the PTENP1 pseudogene regulates PTEN expression, and KRASP1 influences KRAS signaling.^46^ We speculate that MGAT4EP may similarly interact with miRNAs or other regulatory elements targeting MGAT4 family members, indirectly impacting glycosylation-related pathways.

Recent studies have also demonstrated that pseudogenes can affect tumor cell metabolism. For example, certain FTH1 pseudogenes generate small interfering RNAs (siRNAs) that regulate iron metabolism and play crucial roles in tumor cells.^47^ Whether MGAT4EP, as a member of the glycosyltransferase family, influences tumor cell metabolism will require further investigation. Additionally, while the presence of frameshift or premature stop codons in MGAT4EP suggests the unlikeliness of truncated protein production, we cannot entirely exclude the possibility that it could generate functional peptides with biological activity. Validation through proteomics or ribosome profiling will be necessary in future studies.

In conclusion, this study systematically elucidates the significant potential of MGAT4EP as a prognostic marker for breast cancer and provides in-depth insight into its complex mechanisms in breast cancer biology. Our research not only provides a solid experimental foundation for the clinical application of MGAT4EP but also opens new avenues for early diagnosis, precise prognostic assessment, and individualized treatment strategies for breast cancer. However, this study has certain limitations, as it primarily relies on TCGA data alongside some *in vitro* and *in vivo* experiments. Therefore, future research should aim to the clinical sample size and incorporate additional animal models to further validate the specific role of MGAT4EP in breast cancer and actively explore its clinical potential as a therapeutic target.

## Materials and methods

### Cells and reagents

MDA-MB468 cells was obtained from American Type Culture Collection (ATCC, VA, USA). The cell line MB231–4175 with stable expression of luciferase is a kind gift from Dr. Joan Massague (Sloan Kettering Institute).^48^ These cell lines were used for subsequent experiments in this study. GAPDH (2118S) was purchased from Cell Signaling Technology (Danvers, MA, USA). ITGA2 (sc -74,466), MCAM-1 (sc -18,837) were purchased from Santa Cruz Biotechnology (USA). The second antibody used in Western blot were purchased from Cell Signaling Technology.

### Acquisition and preprocessing of TCGA data

To identify genes associated with breast cancer prognosis, transcriptomic and clinical data related to breast cancer were downloaded from the TCGA database (https://portal.gdc.cancer.gov/). Transcriptomic data were normalized using the “limma” R package, with the selection criteria set to *p* < .05 and |logFC| > 1 (Supplementary Figure S1). Differentially expressed genes between normal and tumor tissues were identified. The transcriptomic data containing differentially expressed genes were then merged with clinical information using a Perl script.

### Survival analysis for gene selection

Survival-related genes were identified using the “survival” R package. Kaplan-Meier (KM) values, Hazard Ratios (HR) with 95% confidence intervals, and COX p-values were calculated, with *p* < .05 as the screening criterion.

### ROC curve for target gene prognostic prediction

The prognostic power of the target genes was assessed using the “survival ROC” R package. ROC values greater than 0.6 were considered significant for screening. The “survival,” “survminer,” and “time ROC” R packages were used to jointly analyze the prediction ability of target genes for 1-year, 3-year, and 5-year prognosis, with ROC curves plotted for visualization.

### Differential expression of target gene

The expression of the target gene in normal and tumor tissues was analyzed using the “limma,” “ggplot2,” and “ggpubr” R packages. Differential analysis between groups was performed, and the results were visualized using boxplots.

### Survival analysis for target gene

Using the “limma,” “survival,” and “survminer” R packages, the target gene expression was used to classify samples into high and low expression groups based on median expression values. Differences between high and low expression groups were assessed, and p-values were calculated. Kaplan-Meier survival curves for “Overall Survival (OS)” and “Progression-Free Survival (PFS)” were plotted.

### Clinical correlation of target gene

Patients were grouped according to T/N/M staging: T staging was divided into T1, T2, T3, and T4; N staging into N0 and N+; M staging into M0 and M1; Stage staging into Stage 1, Stage 2/3, and Stage 4. The expression of the target gene across different clinical traits was analyzed using the “limma” and “ggpubr” R packages, with results visualized using boxplots.

Using the “Complex Heatmap” and “limma” R packages, expression data were divided into high and low expression groups based on the median expression value of the target gene. Clinical traits were analyzed for differences between these groups, and significance was assessed, with results visualized in a heatmap.

### Prognostic correlation of target gene

Using the “survival” R package, the target gene and clinical information from TCGA (T, N, M, Age, ER, PR, HER2 and AJCC stage) were integrated for univariate and multivariate COX analysis. The results were presented in a forest plot.

### Predictive model construction

Based univariate and multivariate analysis results, statistically significant variables were incorporated into the analysis to build a predictive model. Since TNM staging and Stage staging overlap, only TNM staging was retained. A Cox proportional hazards model was built using the “survival” package’s coxph function, incorporating clinical and expression data to assess the influence of predictive variables on survival risk.

### Nomogram

A nomogram was generated using the “regplot” R package, based on the Cox model results, to visually display the contribution of each predictive variable to survival risk. Parameters could be customized, and risk scores for each sample were calculated.

### ROC curve

The “time ROC” package was used to calculate and plot time-dependent ROC curves for specific time points, assessing the model’s predictive performance. Multiple curves allowed comparison of predictive accuracy at different time points.

### Calibration curve

Using the “rms” package’s cph and calibrate functions, the Cox model was constructed and calibrated. Calibration curves were plotted to compare predicted survival probabilities with actual survival outcomes.

### Gene enrichment analysis

Samples were divided into high and low expression groups based on the median expression value of the target gene. Differential analysis was conducted using the “limma” R package with *p*-value <.05 and *p*. adjust < 0.05 as criteria. Gene enrichment analysis was carried out using “org.Hs.eg.db,” “clusterProfiler,” “enrichplot,” and “dplyr” R packages.

### siRNA Transfection

Two siRNA sequences targeting MGAT4EP were designed:

MGAT4EP siRNA #1: CCTGATTTCTCATTTCCAT

MGAT4EP siRNA #2: ACTCGTGGGTGCTAATGGA

These were synthesized by Sangon Biotech (Shanghai) Co., Ltd. MDA-MB-468 and MDA231–4175 cells were cultured to a log-phase growth and transfected using Lipofectamine 2000 (Invitrogen, NY, USA).

### RNA-seq

MDA-MB468 and MB231–4175 cells were treated with siRNA to silence MGAT4EP expression. Total RNA was extracted using Trizol, following standard RNA extraction and purification protocols to ensure RNA integrity and quality. The RNA samples were then sent to BGI for high-throughput RNA sequencing (RNA-Seq).

### sh-MGAT4EP construction

The lentivirus sequence was designed as: ACTCGTGGGTGCTAATGGA, and the LV3 (H1/GFP&Puro) vector was used for the virus construction. The virus was prepared by Suzhou Genepharma Co., Ltd. When MB231–4175 cells reached 70–80% confluence, these cells were transfected with lentivirus using Polybrene.

### Flow cytometry

MDA-MB468 and MB231–4175 cells were treated with siRNA to silence MGAT4EP expression. Apoptosis was detected using the Annexin V-FITC/PI Apoptosis Detection Kit (from Wanlai Biological Company). The kit uses the principle that Annexin V binds to phosphatidylserine (PS) exposed on the cell membrane during early apoptosis, combined with propidium iodide (PI) for dual staining, to distinguish between early apoptotic, late apoptotic, and necrotic cells. Flow cytometry was used to analyze the stained cells and assess the effect of siRNA treatment on cell apoptosis.

### RT-qPCR

Primers for MGAT4EP were designed as follows:

MGAT4EP Forward (F): TTGTGGAATGGGAGGTACGC

MGAT4EP Reverse (R): CCCTCCGAAAGATGTCCCAG

Primers were synthesized by Sangon Biotech (Shanghai) Co., Ltd. Total RNA was extracted utilizing the TRIzol reagent (Takara, Dalian, China). Subsequently, cDNA was synthesized with the aid of the HiScript II 1st Strand cDNA Synthesis Kit (Vazyme). For quantitative real-time PCR (qRT-PCR) analysis, the HiScript II One Step qRT-PCR SYBR Green Kit (Vazyme) was employed. Primers of other genes are listed in Supplementary Table 2.

### Western blot

48 hours after siRNA transfection, cells were lysed using RIPA buffer. Proteins were separated by SDS-PAGE at the appropriate concentration and then transferred onto PVDF membranes. Protein bands corresponding to the target molecular weight were cut out and incubated overnight at 4°C with specific primary antibodies. The next day, the membranes were washed and incubated with HRP-conjugated secondary antibodies at room temperature for 1 hour. Protein bands were detected using an enhanced chemiluminescence (ECL) detection kit (Tiangen Biotech, Shanghai, China).

### Scratch assay

When MB231–4175 cells reached the logarithmic growth phase, they were digested with trypsin and counted. Cells were seeded at a density of 8 × 10^4^ cells per well. After 24 hours, siRNA transfection was performed. Following successful transfection for 24 hours, a 0-hour scratch was made using a 200 μL plastic pipette tip to create a linear wound. The cells were then washed with PBS to remove floating cells. Images were captured at 0 and 72 hours to evaluate the effect of si-MGAT4EP on the migration ability of breast cancer cells.

### Transwell assay

After successful siRNA transfection, MDA-MB468 (12 × 10^4^) and MB231–4175 (8 × 10^4^) cells were suspended in serum-free medium at concentrations of 1 × 10^5^ (MDA-MB468) and 2 × 10^4^ (MB231–4175), respectively, and seeded in the upper chamber of a Transwell insert. The lower chamber contained complete medium. After 24 hours, cells were fixed with 4% paraformaldehyde for 15 minutes, followed by staining with crystal violet for 30 minutes. After washing with PBS, images were taken.

### CDX model

To investigate the effect of MGAT4EP on tumor growth, MDA-MB468 cells were cultured to logarithmic phase and infected with sh-MGAT4EP and sh-NC (negative control) viruses. After successful infection, 10^7^ MDA-MB468 cells were subcutaneously injected into the backs of 4–6 week-old NOD-SCID mice. Tumor volume was measured regularly using the formula: V = (π/6) * L * W^2, where L is the tumor’s longest diameter and W is the shortest diameter.

### Lung metastasismodel

MB231–4175 cells, which are fluorescent and have metastatic properties, were cultured to logarithmic phase and infected with sh-MGAT4EP and sh-NC viruses. After successful infection, 10^6^ MDA-MB468 cells were injected via the tail vein into 4–6 week-old nu/nu mice to establish a lung metastasis model. In vivo imaging was used to observe the development of the lung metastasis model and breast cancer lung metastasis.

### Statistics

Statistical analyses were performed using R 4.4.0 and GraphPad software (GraphPad Prism 9.0). The results are presented as the mean ± standard deviation (SD) from at least three independent experiments. Differences between two groups were analyzed using Student’s t-test. Significance levels were set as follows: *p* < .001, ***; *p* < .01, **; *p* < .05, *.

## Supplementary Material

Supplemental Material

## Data Availability

All data produced or analyzed in this study can be found within this article. The datasets used and/or analyzed during the current study are available from the corresponding authors upon reasonable request.
