# Supplementary material for: MGAT4EP promotes tumor progression and serves as a prognostic marker for breast cancer
Source: Cancer Biol Ther. 2025 Mar 11;26(1):2475604. doi: 10.1080/15384047.2025.2475604 (PMC11901376; doi:10.1080/15384047.2025.2475604)
Supplement: Supplemental Material [file KCBT_A_2475604_SM0131.docx]

**MGAT4EP promotes tumor progression and indicator of poor prognosis of breast cancer**

LinZhong^1^, JianfengSu^1^, JieChen^1^, XuchuJin^1^, QuanliangLiu^1^, FengshuJi^2✉^, JingLuo^1✉^, HongWang^3✉^

1. Department of Breast Surgery, Sichuan Academy of Medical Sciences, Sichuan Provincial People's Hospital, Chengdu, Sichuan, 610072, China
2. Department of General Surgery, Zhujiang Hospital of Southern Medical University, Guangzhou, Guangdong, 510006, China
3. School of Pharmacy, Sun Yat-sen University, Guangzhou, Guangdong, 510080, China

✉e-mail: [jishu_feng@163.com (Shu](mailto:jishu_feng@163.com(fengshu)fengJi), [luckyluojy@163.com (JingLuo),](mailto:luckyluojy@163.com,) [wangh559@mail2.sysu.edu.cn](mailto:wangh559@mail2.sysu.edu.cn) (HongWang)

**Figure S1**

**
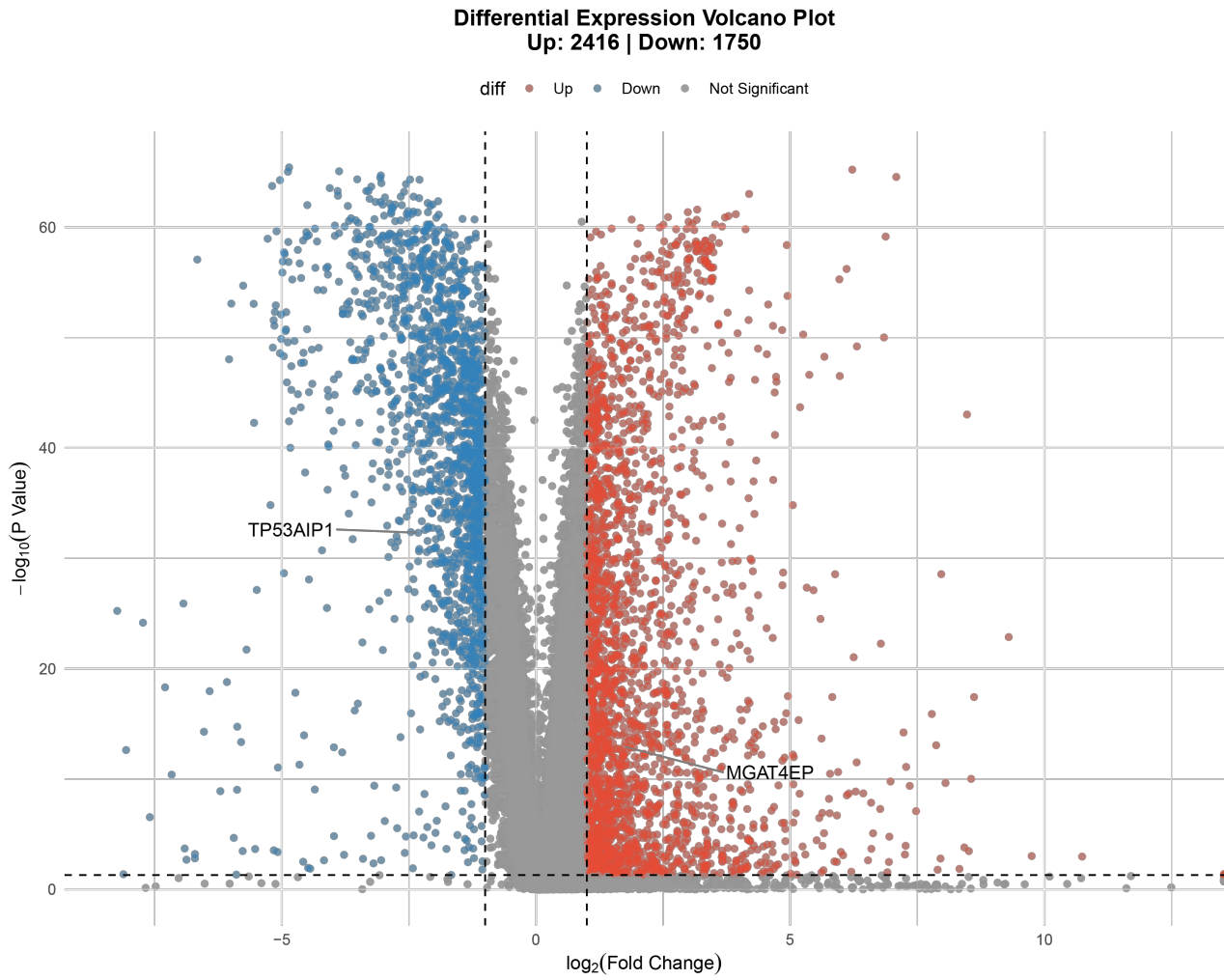
**

**Supplementary Figure 1. Differential gene analysis between breast cancer and normal tissues.**

Differential gene analysis between breast cancer and normal tissues with screening criteria of *p* < 0.05 and |logFC| > 1, presented as a volcano plot.

**Figure S2**

**
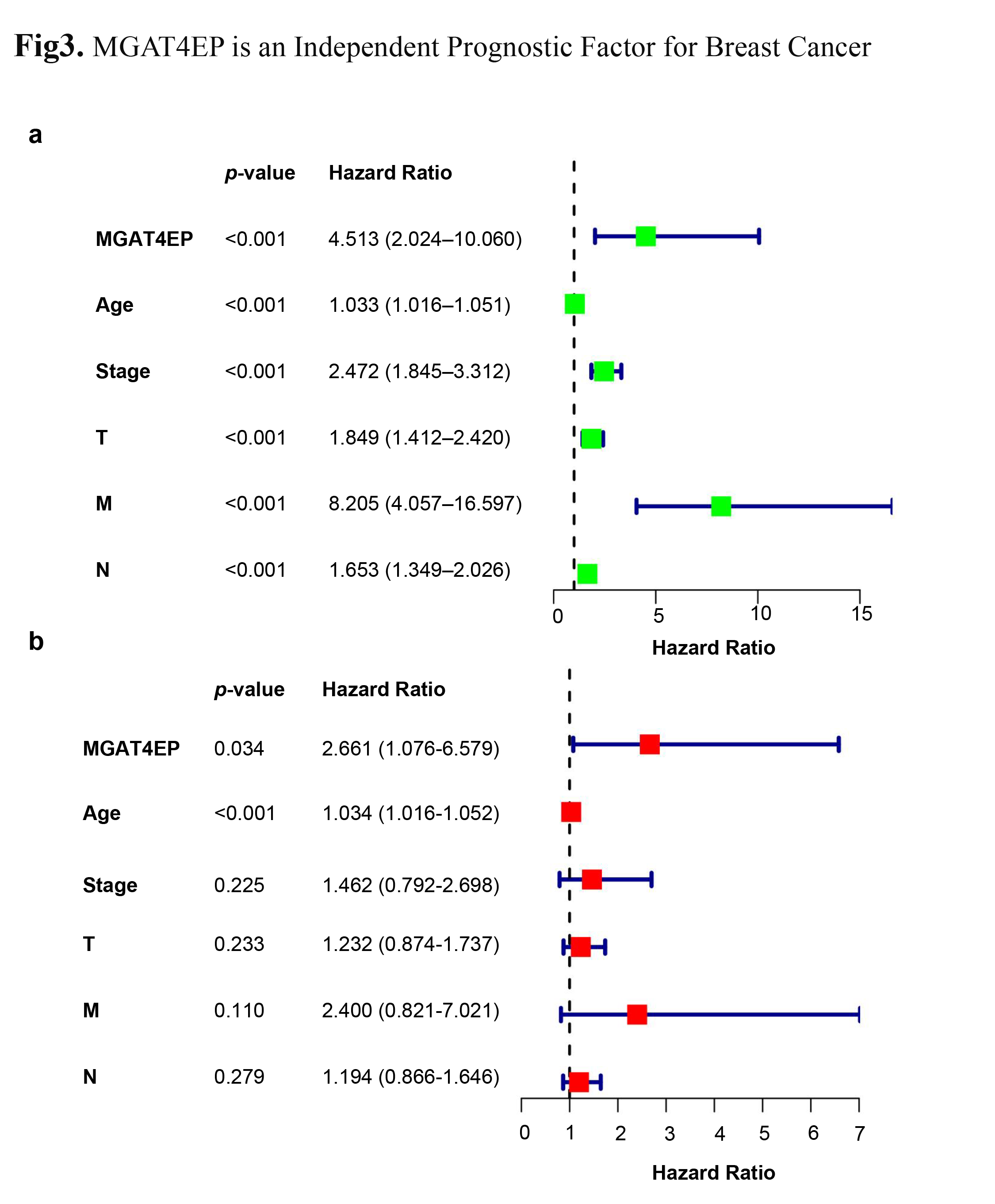
**

**Supplementary Figure 2. MGAT4EP as an independent prognostic factor for breast cancer based on combined analysis of TNM, age, and AJCC stage.**

a. Univariate analysis of MGAT4EP combined with age, AJCC stage, tumor size, lymph node metastasis, and distant metastasis, presented as a forest plot.
b. Multivariate analysis of MGAT4EP combined with age, AJCC stage, tumor size, lymph node metastasis, and distant metastasis, also presented as a forest plot. * *p* < 0.05, ***p* < 0.01, ****p* < 0.001.

**Figure S3**

**
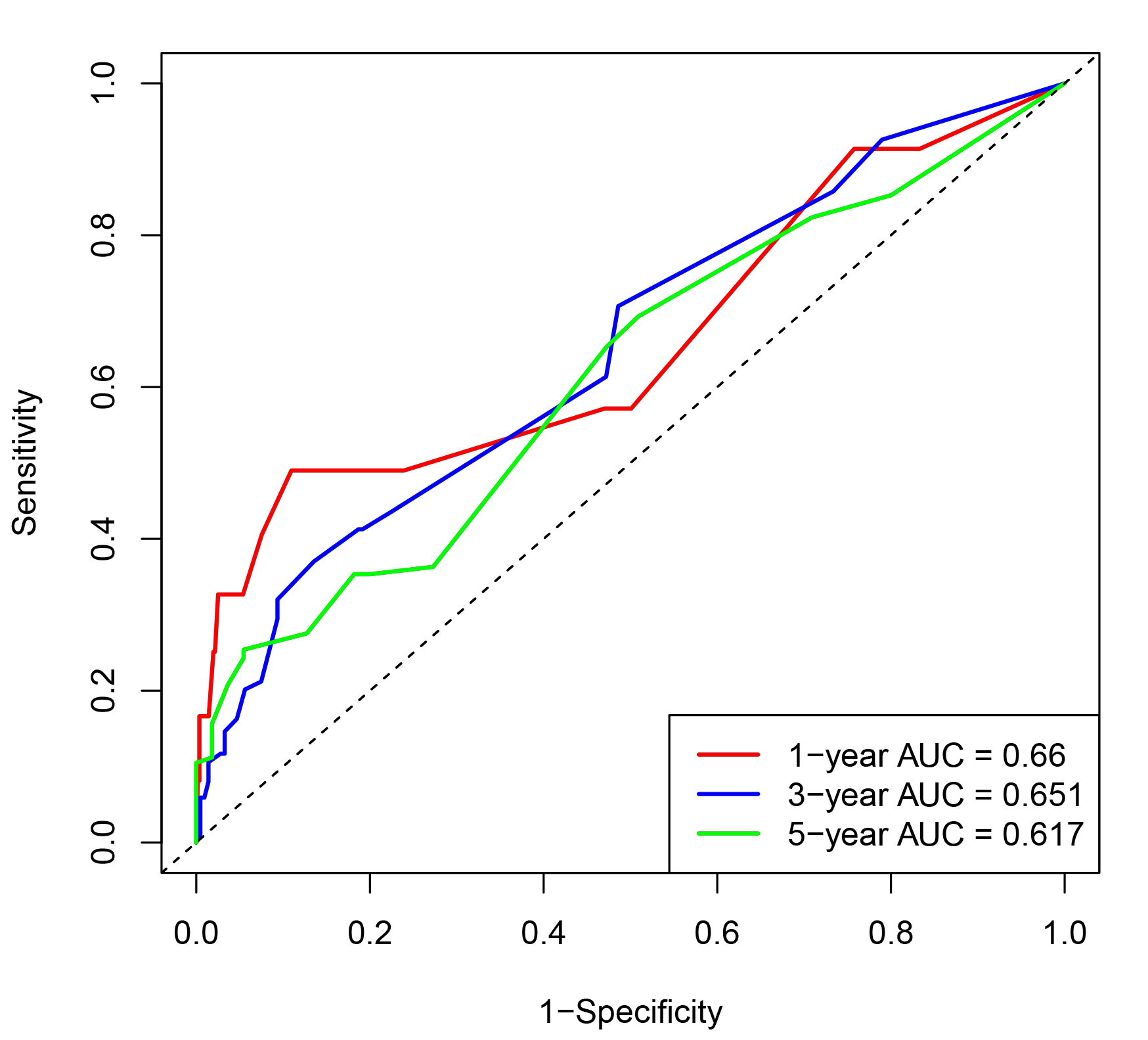
**

**Supplementary Figure 3. ROC curve analysis for evaluation of breast cancer prognosis using the TNM staging system.**

**
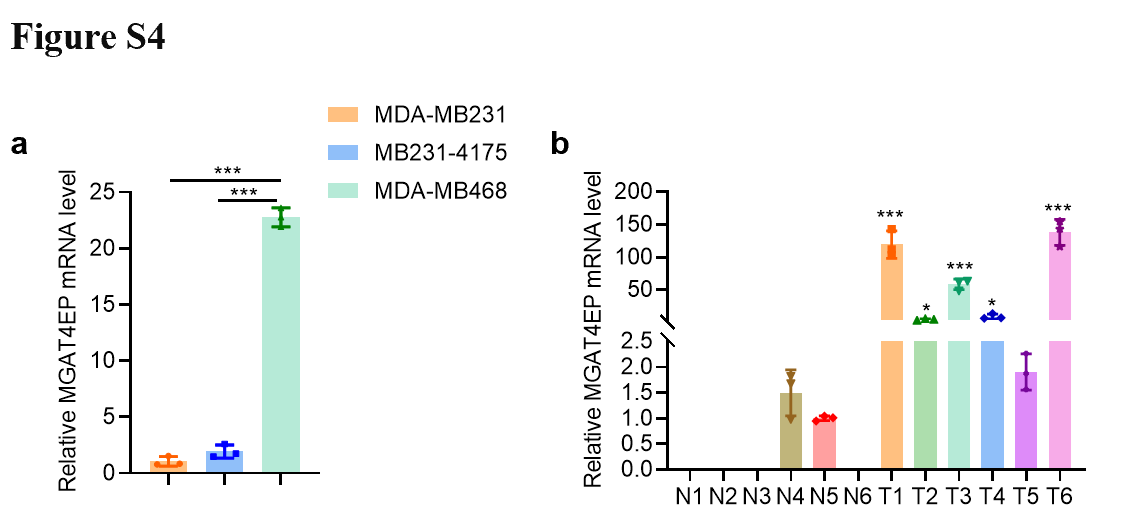
**

**Supplementary Figure 4. The Expression of MGAT4EP is Closely Related to the Clinical Characteristics of Breast Cancer.**

a-b. qRT-PCR analysis of MGAT4EP gene in TMBC cell lines and tissue. *n* = 3 biological replicates. Data shown above are the mean ± SD. * *p* < 0.05, ***p* < 0.01, ****p* < 0.001.

**
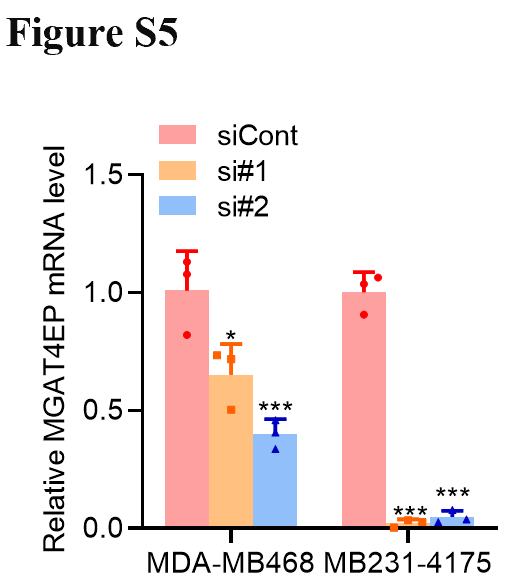
**

**Supplementary Figure 5. MGAT4EP affects the progression of breast cancer by regulating apoptotic pathways.**

qRT-PCR analysis of MGAT4EP gene in MDA-MB468 and MB231-4175 cells after transfected with vehicle or MGAT4EP siRNA for 2 days. *n* = 3 biological replicates. Data shown above are the mean ± SD. * *p* < 0.05, ***p* < 0.01, ****p* < 0.001.


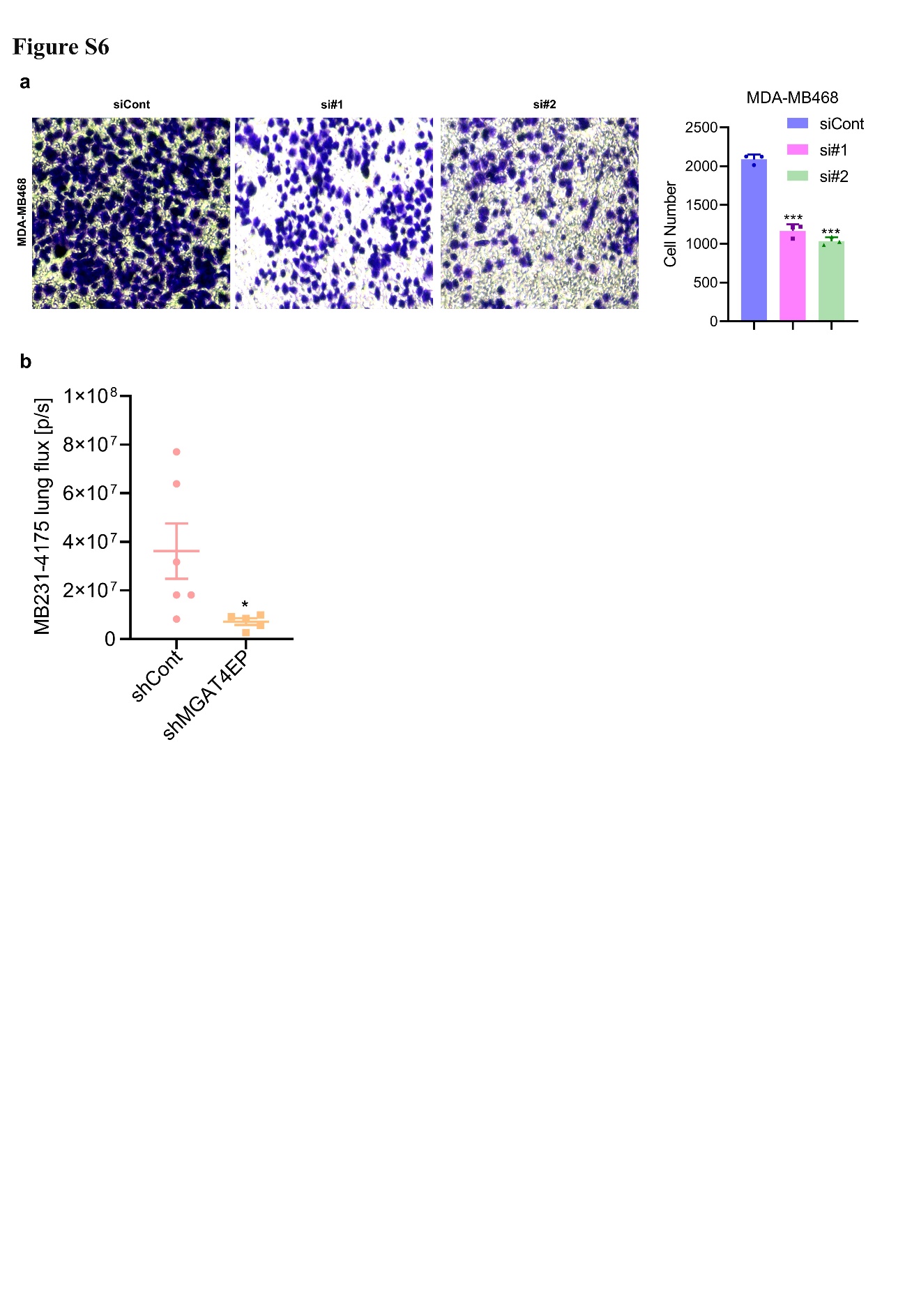


**Supplementary Figure 6. MGAT4EP promotes breast cancer metastasis through the focal adhesion pathway.**

a. Transwell assay exploring the effect of MGAT4EP silencing on the invasion ability of MDA-MB468 cells. *n* = 3 biological replicates. Data shown above are the mean ± SD. * *p* < 0.05, ***p* < 0.01, ****p* < 0.001.

b. Luciferase fluorescence signal intensity of lung from mice after transfected with vehicle or MGAT4EP shRNA for 21 days (*n* = 6 mice per group). Data shown above are the mean ± SEM. * *p* < 0.05.

**Supplementary Table 1. TP53AIP1 and MGAT4EP are key genes that influence breast cancer prognosis.**

***** KM ( Kaplan-Meier ), HR(Hazard Ratio).

**Supplementary table 2. Primers for qPCR**

| **Primers for qPCR** |  |
| --- | --- |
| β-actin F | GAGAAAATCTGGCACCACACC |
| β-actin R | ATACCCCTCGTAGATGGGCAC |
| TGM2-F | CACCCACACCTACAAATACCC |
| TGM2-R | AAGATCCCATTGTAGCTGACG |
| MCAM-F | TTGCATGACCTGAAACGGGA |
| MCAM-R | GTCTTGTTCACTTGCCGTGC |
| GJA1-F | AAAGAGCGACCCTTACCATG |
| GJA1-R | GCCCCATTCGATTTTGTTCTG |
| ITGA2-F | GCCCTTCCCTCATAGATGTTG |
| ITGA2-R | TTGGGATGTCTGGGATGTTG |
| CAV1-F | CCTTCCTCAGTTCCCTTAAAGC |
| CAV1-R | TGTAGATGTTGCCCTGTTCC |
